# Elastic Elements in a Wrist Prosthesis for Drumming Reduce Muscular Effort, but Increase Imprecision and Perceived Stress

**DOI:** 10.3389/fnbot.2018.00009

**Published:** 2018-03-19

**Authors:** Georg Stillfried, Johannes Stepper, Hannah Neppl, Jörn Vogel, Hannes Höppner

**Affiliations:** ^1^German Aerospace Center (DLR), Institute of Robotics and Mechatronics, Wessling, Germany; ^2^Statistisches Beratungslabor, Ludwig-Maximilians-Universität, Munich, Germany

**Keywords:** variable-stiffness actuators, series-elastic actuators, wrist prosthetics, drumming, cyclical tasks, energy storage

## Abstract

Recently, progress has been made in the development of mechanical joints with variable intrinsic stiffness, opening up the search for application areas of such variable-stiffness joints. By varying the stiffness of its joints, the resonant frequency of a system can be tuned to perform cyclical tasks most energy-efficiently, making the variable-stiffness joint a candidate element for an advanced prosthetic device specifically designed for the cyclical task of drumming. A prerequisite for a successful variable-stiffness drumming prosthesis is the ability of human drummers to profitably employ different stiffness levels for playing different beats. In this pilot study, 29 able-bodied subjects (20 drumming novices and 9 experts) wear a cuff on the forearm, to which a drumstick is connected using changeable adapters, consisting of several leaf springs with different stiffness and one maximally stiff connection element. The subjects are asked to play simple regular drum beats at different frequencies, one of which is the resonant frequency of the adapter-drumstick system. The subject's performance of each drumming task is rated in terms of accuracy and precision, and the effort is measured using questionnaires for the perceived stress as well as electromyography (EMG) for the muscular activity. The experiments show that using springs instead of the stiff connection leads to lower muscular activity, indicating that humans are able to use the energy-storing capabilities of the springs, or that muscular activity is reduced due to the lower mass of the springs. However, the perceived stress is increased and the novices' performance lowered, possibly due to a higher cerebral load for controlling the elastic system. The hypothesis that “matching the resonant frequency of the spring-drumstick system to the desired frequency leads to better performance and lower effort” is not confirmed. Possible explanations are discussed. In conclusion, a series-elastic element appears to lower the muscular effort of drumming, while a stiff connection appears to minimize the mental load and has a positive effect on the performance of drumming novices.

## 1. Introduction

Series-elastic actuators (SEAs) as well as variable-stiffness actuators (VSAs) have been recently introduced into robotics (Vanderborght et al., 2013) to address current problems of robotic arms with torque sensing and actively controlled compliance (Albu-Schäffer and Hirzinger, 2002; Albu-Schäffer et al., 2007).

Compared to actively compliant actuators, VSAs and SEAs yield several advantages. They can (a) reduce impacts of collisions for motors and gears (Grebenstein et al., 2011), (b) increase dynamic capabilities by allowing to frequently store elastic energy, and (c) embody desired behavior (Visser et al., 2011).

SEAs include an elastic element to decouple motor and link-side positions. VSAs are additionally able to tune this elasticity by using a second motor. This intrinsic elasticity inherently dominates the orientation of favorable compliant directions for multi-joint robotic arms and determines their resonance modes. While this inherent behavior and the more complex dynamics of SEAs and VSAs increase the complexity of controlling arbitrary behavior, they allow reducing the control effort (Visser et al., 2011) and energy consumption of dominating tasks if these are accounted for in the design process of the robotic system.

Currently, we are searching for tasks that can exploit the full potential of SEAs and VSAs. Using joint elasticity to store potential energy appears particularly promising in the reversal points of cyclical tasks. They can be performed energy-efficiently at different speeds if the system performing the task contains VSAs, because the resonance frequency of the system can be tuned by changing the stiffness values of its joints. An example for a cyclical task involving the upper limbs is drumming. It has already been proven that the drum roll frequency of a robotic drummer can be controlled by varying the robot's passive stiffness (Hajian et al., 1997; Hajian, 1997). In the same study, the authors “…present evidence that drummers vary the stiffness of their hands to control the bounce frequency…”. They showed that healthy subjects increase grip force and—since grip stiffness and grip force go hand in hand (Höppner et al., 2011)—naturally grip stiffness as well, to increase the drumming speed during double-stroke drum rolls.

Studying human impedance is an active field of research that frequently inspires robotics. Studies in human wrist stiffness show that wrist stiffness is increased in the presence of unstable loads and, similar to grip stiffness, increases linearly with the applied load (De Serres and Milner, 1991). Additionally, active control, namely the stretch reflex, considerably assists the wrist in a fast return of the limb after displacement (Sinkjær and Hayashi, 1989). Moreover, it is essential to note that wrist stiffness and damping increase as finger force increases, e.g., applied in a tripod-grasp (Kuchenbecker et al., 2003). In general, the human hand can be considered as a VSA rather than a SEA and is able to decouple stiffness from its linear increase with force using cocontraction of antagonistic pairs of muscles (Höppner et al., 2017).

**Figure 1:**
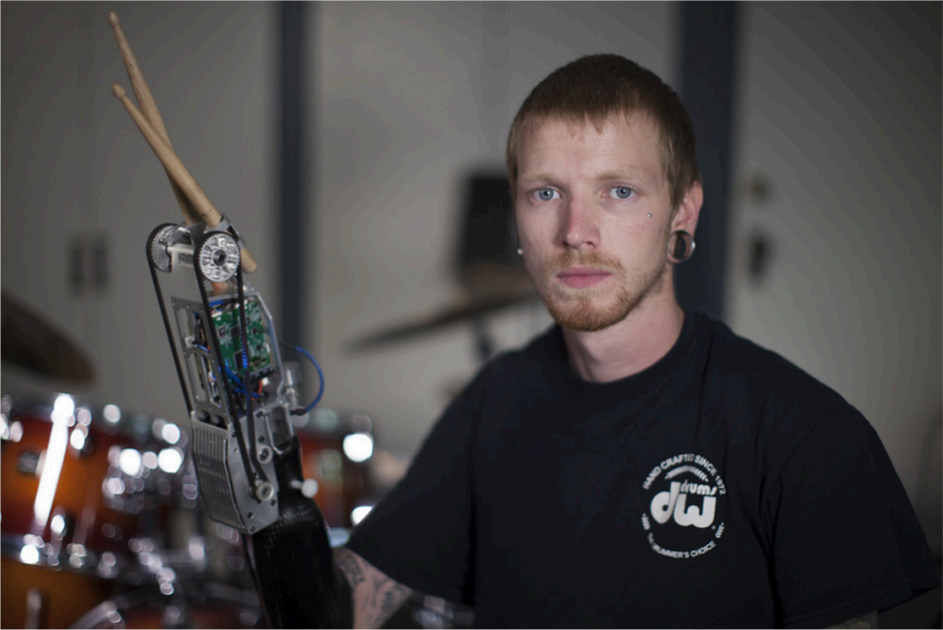
Jason Barnes— The drummer is wearing a prosthesis with actively controlled drumsticks on the stump. The rebound of the drumstick is tuned by using a DC motor in a variable-impedance shared-control framework. Picture by Lwp Kommunikáció (2014). License: CC-BY 2.0.

There are cases in which drummers lose their wrist due to accident or illness, and cases in which persons with congenitally absent parts of the arm would like to play the drums. In these cases, the drumstick has to be attached to the remaining part of the arm (except if they use only the intact arm, or compensate using their legs). A prosthetic drumstick holder is commercially available, including an elastic element, but without variable stiffness (TRS Inc., 2017). Increasing the level of technological sophistication, researchers from Georgia Tech in Atlanta equipped drummer Jason Barnes' right arm, which has suffered a transradial amputation, with a myo-electrically controlled robotic prosthesis (see Figure 1). The prosthesis was equipped with a drumstick and, by using a DC motor in a variable-impedance shared-control framework, the rebound of the drumstick after initial impact was controlled (Bretan et al., 2016). However, no variable mechanical elasticity was implemented.

If variable mechanical elasticity was implemented, it could be tuned by either a myo-electrical or velocity-proportional control. Myo-electrical control of position and stiffness of a VSA was investigated by Hocaoglu and Patoglu (2012), while this study did not investigate energetic interactions with the environment. Ajoudani et al. (2012) and Godfrey et al. (2013) tuned an actively controlled compliance—also called *apparent stiffness* in biomechanics (Latash and Zatsiorsky, 1993)—using electromyography (EMG) and were able to show general advantages of compliance during object handling and manipulation. However, benefits of mechanical elasticity as energy storage in reversal points during cyclic movements were not the focus of these studies.

Fujii et al. (2009) investigated the drumming performance of unimpaired non-drummers, ordinary drummers and the world's fastest drummer. They found a maximal single-stroke drumming frequency for non-drummers and ordinary drummers alike of 6–7 Hz, while the world's fastest drummer was able to play beats of 10 Hz. We use the values of non-drummers and ordinary drummers in our study to limit the range of drumming frequencies that subjects are supposed to play.

Based on the double motivation of searching for an application of VSAs and trying to improve prosthetic drumming experience, we want to investigate in a user study whether introducing inherent elasticity into a wrist prosthesis might be beneficial for drumming and want to answer the question: *Can drummers take advantage of variable stiffness in a prosthetic wrist?*

It seems clear that stiffness is increased in healthy drummers to increase drumming speed (Hajian et al., 1997; Hajian, 1997). But it remains unclear whether this coupling between stiffness and bounce frequency is the result of an increase in grip strength or whether stiffness *per se* is the primarily optimized parameter, for example to enhance exploitation of energy storing capabilities. Moreover, it is known that the motor control of humans is able to achieve and stabilize coordinated cyclic movements even in the presence of strong dynamic non-linearities (Lakatos et al., 2014). But can we exploit benefits of artificial elastic joints to reduce the required energy and to increase comfort? Compared to a rigid attachment, an elastic attachment might not only reduce the required amount of energy but would be able to absorb the impact of the drum on the drumstick.

Furthermore, it is of interest whether a prosthetic wrist with a fixed elasticity is sufficient, or whether changing the stiffness during drumming is beneficial. Fixed elasticity is implemented using SEAs and variable stiffness using VSAs, which has a serious commercial background, since VSAs require the implementation of an additional drive.

Hence, the main purpose of this study is to investigate which of the following prosthetic wrist types enables best drumming performance and provides the most comfortable playing experience: a stiff connection, a spring with a fixed stiffness, or an elastic adapter with variable stiffness.

To simplify the preparation of the experiment, instead of a joint with continuously variable stiffness, discrete elastic elements with different stiffness values are used.

**Figure 2:**
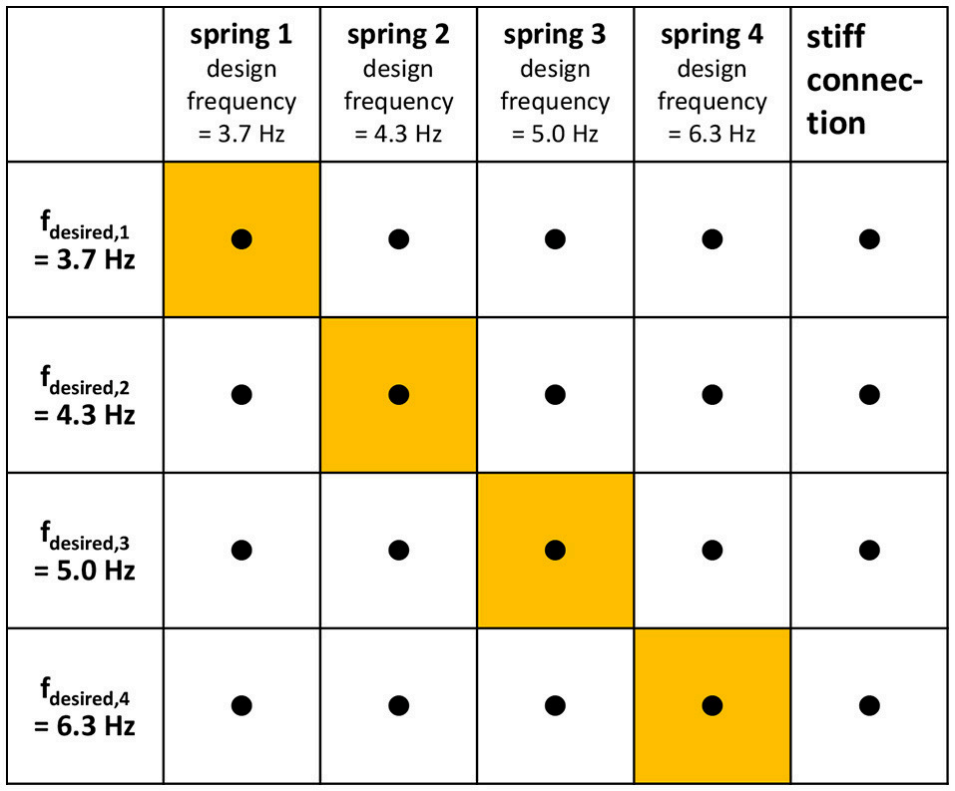
Trials— Combination matrix of the factors “desired frequency” and “adapter type.” Each dot represents one trial, i.e., the subjects are asked to play all combinations. Note that the desired frequencies are chosen so that they correspond to the design frequencies of the four springs. The variable-stiffness strategy of this study, where the desired frequency is matched by the design frequency, corresponds to the highlighted diagonal of the matrix.

Using a velocity-proportional control scheme, the discretely variable stiffness is chosen so that the design frequency, which is an approximation of the resonant frequency of the system, matches the desired playing frequency. We call this control scheme “diagonal-type variable stiffness,” because it corresponds to the diagonal of the combination matrix of desired frequency and variable stiffness (Figure 2).

Our hypotheses are (a) that subjects play best with diagonal-type variable stiffness, and, (b) that experts are better in taking benefit of the variable stiffness, since they are familiar with the general task of drumming, which probably frees their mental capacities for adapting to the new device.

## 2. Materials and methods

This study involved 20 unimpaired drumming novices and 9 unimpaired drumming experts who wore a cuff on the forearm, to which a drumstick was attached using stiff and elastic adapters. Hence, the subjects' intact wrists were not used but replaced with an experimental prosthetic attachment, simulating the situation of a missing hand. The subjects were asked to play all combinations of adapter types and desired frequencies shown in the combination matrix in Figure 2. The desired frequencies ranged from typical beats of popular music (3–4 Hz) to the maximum single stroke frequency that an average drumming novice can reach according to Fujii et al. (2009) (6–7 Hz). Measurements of muscular activity and a questionnaire were used to assess each subject's stress level, while the inaccuracy and imprecision of the beat were used to judge the quality of the drumming.

### 2.1. Experiment setup

**Figure 3:**
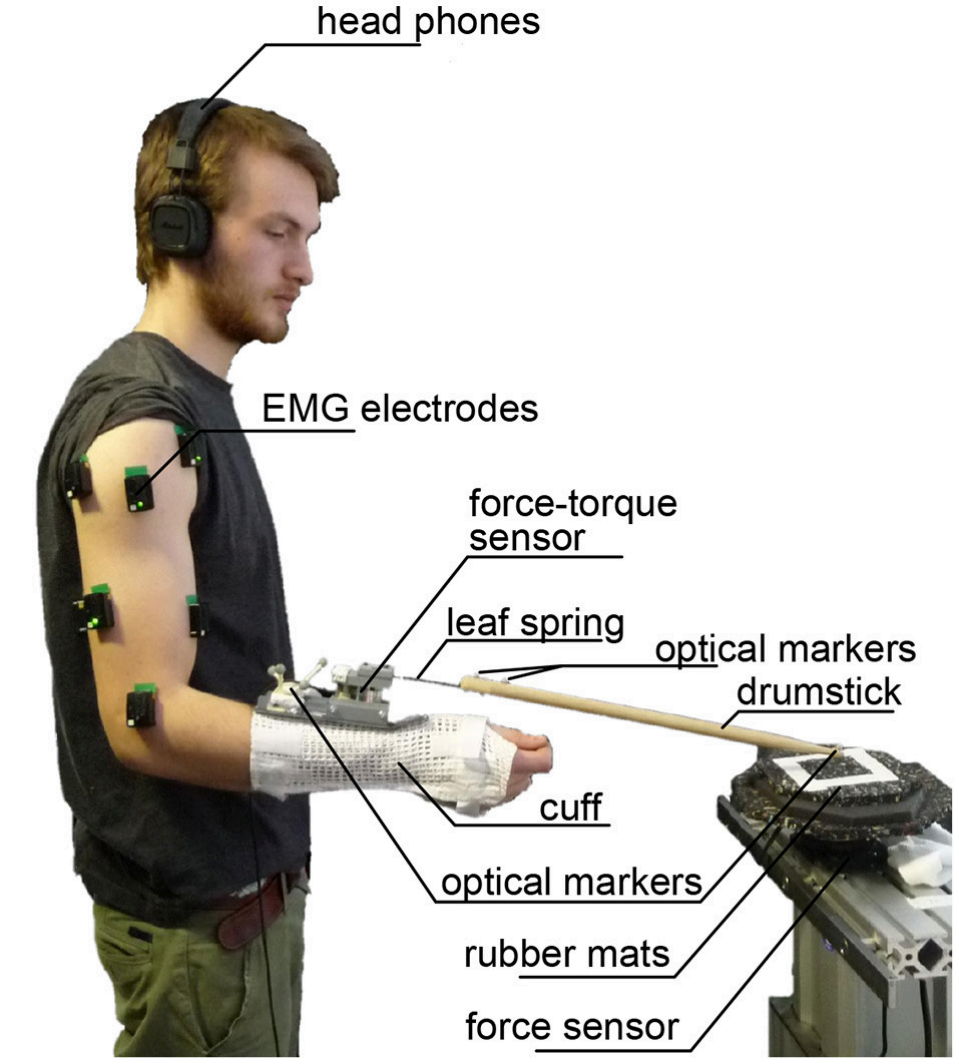
Experimental Setup— Subjects were equipped with a cuff with the attached drumstick, electrodes for measuring muscular activity of respective muscles, as well as markers for optical tracking. Force-torque sensors were placed between drumstick and cuff as well as on the drumming plate. Subjects were given head phones with a metronome beat with the desired drumming frequency. This picture shows one of the researchers and co-authors of this study, who has provided written informed consent to this publication of his image.

The experiment setup is shown in Figure 3. The experiment took place in a space secluded by curtains from the rest of the room in order to avoid distraction of the subject.

**Figure 4:**
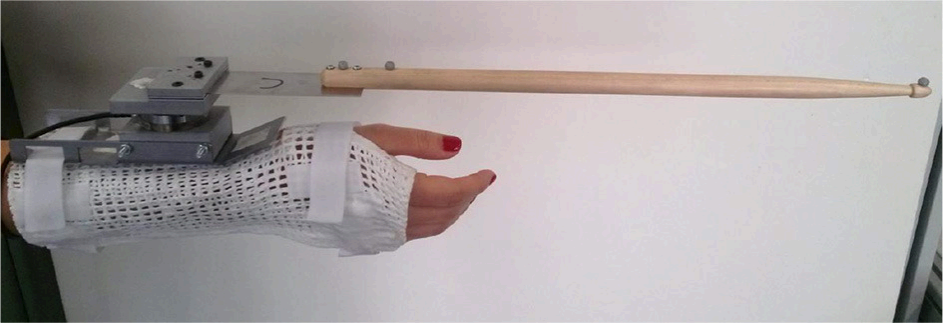
Drumming cuff with a soft spring— The force-torque sensor as well as the markers for optical tracking of the drumstick can be seen. The cuff is designed such that it prevents the wrist from extension/flexion and radial/ulnar deviation.

**Figure 5:**
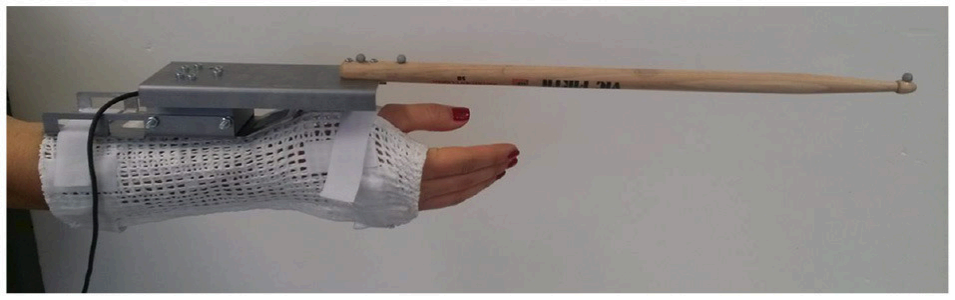
Drumming cuff with the stiff connection.

The subject wore a cuff constraining movements of the wrist (extension/flexion and radial/ulnar deviation). The cuff was coupled to the drumstick via a changeable adapter. The set of changeable adapters consisted of four leaf springs with different stiffness values (see exemplary spring in Figure 4) and one maximally stiff connection element (see Figure 5). The adapter was approximately aligned in parallel to the plane of the radial and ulnar deviation of the wrist.

Between spring and cuff a force-torque sensor ATI Mini45 SI-290-10 was placed (measurement range: *F*_x/y_ = ±290 N, *F*_z_ = ±580 N, *T*_x/y/z_ = ±10 Nm; resolution: Δ*F*_x/y/z_ = ±1/8 N, Δ*T*_x/y_ = ±1/376 Nm, Δ*T*_z_ = ±1/752 Nm). This sensor was used for determining the design frequency of each adapter element, which corresponds to the resonant frequency of the whole cuff including force-torque sensor, spring and drumstick. For this, the cuff was fixed in a bench vise, and the drumstick was jolted into free oscillation. From the force-torque sensor data, the frequencies of the oscillation were found to be at 3.7, 4.4, 5, and 6.3 Hz for the springs and at 27 Hz for the stiff connection. The force sensor remained between spring and cuff during the experiments to avoid changing the design frequencies. The springs were approximately 70–90 g lighter than the stiff connection, leading to a reduction of the moment of inertia around the elbow flexion axis of about 5–6%.

The drum strokes were recorded by a JR3 90M31A3 force-torque sensor fixed on a table (measurement range: *F*_x/y_ = ±200 N, *F*_z_ = ±400 N, *T*_x/y/z_ = ±20 Nm; resolution: Δ*F*_x/y_ = ±0.050 N, Δ*F*_z_ = ±0.10 N, Δ*T*_x/y/z_ = ±0.005 Nm). The sensor was covered with rubber mats for damping the noise and making the drumming more comfortable (see Figure 3). Note that this damping influenced the peak forces, but not the time of impact, the latter being relevant for evaluating drumming imprecision and inaccuracy. The height of the table was adjusted to the subject's height.

The force-torque sensor data was low-pass filtered using a Butterworth filter with 100 Hz cutoff frequency. The time between strokes was determined using the peak detection function of Matlab on the filtered vertical force data, with the parameters minimum peak height set to 0.5 times the 95th percentile of the filtered data set and minimum peak distance set to 0.5 times the desired time between strokes. Times between strokes that exceeded 1.5 times the median time between strokes were counted as missed strokes or pauses and discarded.

For possible later reference, the positions of cuff and drumstick were continuously monitored through optical tracking.

The desired frequency was given by the beat of a metronome via head phones, which was also recorded for later reference.

We used EMG electrodes for measuring the muscular activity of subjects. The surface electrodes of the *Delsys Trigno Wireless System* have an internal amplification of 1 kV/V and provide an analog signal at 4 kHz with a constant delay of 48 ms. These electrodes comply with the requirements put forth by the Medical Device Directive 93/42/EEC, and we complied with their intended use.

We measured the muscular activity of 8 muscles involved in shoulder and elbow movements: biceps brachii (elbow and shoulder flexion, shoulder abduction), pectoralis major (humerus adduction), deltoideus posterior (shoulder extension), deltoideus medius (shoulder abduction), deltoideus anterior (shoulder flexion), anconeus (elbow extension), triceps brachii long head (elbow and shoulder extension), and triceps brachii lateral head (elbow extension). The mean of the EMG values over all trials of each electrode was subtracted subject-wise to remove any constant DC offset. Furthermore, after subtracting the DC offset, the EMG values of each electrode were normalized subject-wise by dividing by the RMS value over all trials. This eliminates differences in electrodes due to location-specific tissue resistance and gives the variations between trials of the signal of each electrode the same weights.

Additionally, the measurement setup consisted of a host computer running Linux, a real-time target computer running VxWorks and a Windows computer. The real-time computer ran the software (developed using Matlab/Simulink) to read out the force-torque and EMG sensors at 1 kHz. The marker positions were recorded by the Windows computer and transferred to the Linux host using the DLR communication protocol *aRDnet* (Bäuml and Hirzinger, 2008).

### 2.2. Study design and experiment session protocol

A total of 29 healthy subjects, 25 male and 4 female, all right-handed and initially fully naive to the experiment, performed the experimental protocol as described below. 9 out of the 29 subjects had at least 1 year of drumming experience and were therefore considered as experts, and the other 20 were counted as novices. All subjects participated voluntarily and gave written informed consent to the procedures, which were conducted in partial accordance with the principles of the Helsinki agreement (non-conformity concerns the point B-16 of the 59th World Medical Association Declaration of Helsinki, Seoul, October 2008: no physician supervised the experiments). Approval was received from the works council of the German Aerospace Center, as well as its institutional board for data privacy ASDA; the collection and processing of experimental data were approved by both committees. For all subjects and experiments the right hand was used, which was restricted by the design of the cuff. Subjects stood upright in all experimental conditions.

The experiment session lasted between 20 and 25 min per subject. At the beginning of each experiment session, the participant was instructed about the experiment using a standardized presentation.

The subjects were asked to play all 20 trials consisting of the combinations of adapter types and desired frequencies shown in the combination matrix in Figure 2. During the trials, subjects were observed and asked to keep the orientation of the leaf spring so that the drumming motion is in the direction of its minimal stiffness. In order to prevent effects of learning or fatigue, the combinations were given to them in a block-randomized order: the adapter types were randomized, and within each adapter type, the desired frequencies were randomized.

Before each trial, subjects had the possibility to get used to the current combination of desired frequency and adapter type within a time of 10 s, followed by a phase of 15 s of collecting drumming data. The 10 s of training data were not recorded. After each trial, subjects were asked to fill in a questionnaire about the combined physical and mental stress level that they felt during playing at the respective combination of desired frequency and adapter type. The question was “How high was the perceived stress while playing of each frequency, with respect to physical and mental effort” 1: very low, 8: very high^1^.

Before and after the trials, base noise EMG during rest of the arm was measured, as well as EMG and maximum force during maximum voluntary contraction in a lifting-up and a pushing-down task. This was used for checking whether the signals look plausible. Unfortunately, for unknown reasons, the electrodes of pectoralis major and anconeus showed very noisy signals for many subjects. We therefore discarded the results of these two electrodes.

### 2.3. Statistical design

To answer our research questions and evaluate our hypotheses, we predefined four outcome measures, which are gathered for each subject and trial:

- The inaccuracy, i.e., the difference between the desired time interval and the mean played time interval between two drum strokes, which tells how well the desired frequency could be met; a lower inaccuracy means a better performance:
  (1) $$y_{\text{inaccuracy}}=\left|\frac{1}{f_{\text{desired}}}-\frac{1}{n_{\text{strokes}}}\sum_{s=1}^{n_{\text{strokes}}}\left(T_{\text{played},s}\right)\right|;$$
- the imprecision, i.e., the standard deviation of the time between two strokes, which tells how evenly the frequency was played; a lower imprecision means a better performance:
  (2) $$y_{\text{imprecision}}=\underset{s\in \left\{1,\ldots n_{\text{strokes}}\right\}}{\text{SD}}(T_{\text{played},s});$$
- the perceived stress consisting of physical and mental effort, i.e., the result of the questionnaire; lower stress means higher comfort:
  (3) $$y_{\text{perceived}\_\text{stress}}=\text{questionnaire}\_\text{entry};\text{ and}$$
- the measured muscular activity, i.e., the mean normalized root-mean-square (RMS) EMG signals, where normalizing means subtracting the per-electrode DC offset and dividing by the per-electrode RMS of the signals of all trials; again, a lower muscular activity means a higher comfort:
  (4) $$\text{DC}\_{\text{offset}}_{e}=\frac{1}{n_{\text{trials}}\;n_{\text{samples}}}\sum_{l=1}^{n_{\text{trials}}}\sum_{t=1}^{n_{\text{samples}}}{\text{EMG}}_{elt}$$
  (5) $${\text{EMG}}_{\text{RMS},\text{all},e}=\sqrt{\frac{1}{n_{\text{trials}}\;n_{\text{samples}}}\sum_{l=1}^{n_{\text{trials}}}\sum_{t=1}^{n_{\text{samples}}}{\left({\text{EMG}}_{elt}-\text{DC}\_{\text{offset}}_{e}\right)}^{2}}$$
  (6) $$y_{\text{muscular}\_\text{activity},l}=\frac{1}{n_{\text{electrodes}}}\sum_{e=1}^{n_{\text{electrodes}}}\sqrt{\frac{1}{n_{\text{samples}}}\sum_{t=1}^{n_{\text{samples}}}{\left(\frac{{\text{EMG}}_{elt}-\text{DC}\_{\text{offset}}_{e}}{{\text{EMG}}_{\text{RMS},\text{all},e}}\right)}^{2}},$$

where EMG_*elt*_ is the measured EMG signal of electrode *e* at time sample *t* in trial *l*, with *n*_electrodes_ = 6, *n*_trials_ = 20 and *n*_samples_ = 3,000.

To statistically analyze the results we built and applied a mixed-effects regression model with fixed and random effects. It allowed to directly include the two hypotheses as fixed effects into our model. The formula of the mixed-effects model is:

(7) $$\begin{array}{l} y_{ijklmn}=\beta_{0}+\beta_{\text{adapter\_type},i}+\beta_{\text{desired\_frequency},j}+\beta_{expert\text{\_}status,k}\\ +\beta_{\text{diagonal},l}+\beta_{\text{diagonal}\times expert\text{\_}status,kl}+\beta_{\text{adapter\_type}\times expert\text{\_}status,ik}\\ +m\beta_{trial\text{\_}number}+\epsilon_{subject,n}+\epsilon_{mn}, \end{array}$$

where *y*_*ijklmn*_ is the response variable, i.e., any of the four above-mentioned outcome measures, *i* is the adapter type, *j* is the desired frequency, *k* is the expert status, *l* is the diagonal status, which is 1 if the combination of adapter type and desired frequency is on the diagonal and 0 otherwise, *m* is the within-subject trial number, *n* is the subject number, β_0_ is the intercept, which is a constant term, β_adapter_type,*i*_ is the fixed effect of the adapter type, β_desired_frequency,*j*_ is the fixed effect of the desired frequency, β_expert_status,*k*_ is the fixed effect of the expert status, β_diagonal,*l*_ is the fixed effect of playing on the diagonal, β_diagonal×expert_status,*kl*_ is the fixed effect of the interaction between the expert status and playing on the diagonal, β_adapter_type×expert_status,*ik*_ is the fixed effect of the interaction between expert status and adapter type, *m*β_trial_number_ is the trial-number-dependent fixed effect of learning or fatigue, ϵ_subject,*n*_ is the subject-specific random effect and ϵ_*mn*_ is the residual random error. The random effects are assumed to follow normal distributions as follows:

(8) $$\begin{array}{l} \epsilon_{mn}\overset{i.i.\text{d}.}{\sim }N\left(0,\sigma^{2}\right)\text{and} \end{array}$$

(9) $$\begin{array}{l} \epsilon_{subject,m}\overset{i.i.\text{d}.}{\sim }N\left(0,\tau^{2}\right). \end{array}$$

The factors adapter type, desired frequency, expert status and diagonal status can assume the levels shown in Table 1. The zero level of each factor is taken as the reference configuration, for which all categorical βs are zero.

**Table 1:** Levels of the factors of the mixed model in Equation (7).

***i***	**Adapter type**	***j***	**Desired frequency**	***k***	**Expert status**	***l***	**Diagonal status**
0	Stiff connection	0	3.7 Hz	0	Expert	0	Off the diagonal
1	Spring 1	1	4.4 Hz	1	Novice	1	On the diagonal
2	Spring 2	2	5.0 Hz				
3	Spring 3	3	6.3 Hz				
4	Spring 4						

The parameters of the mixed models were fitted to the measured outcome measures using the *lmer* function of the *lme4* library (Bates et al., 2015) of the R statistics software (R Core Team, 2015). It turned out that for inaccuracy and imprecision, the distribution of the residuals ϵ_*mn*_ could be made much more similar to the assumed normal distribution by transforming them with natural logarithms of their values in s:

(10) $$\begin{array}{l} y_{\text{ln}(\text{inaccuracy})}=\ln\left(\left|\frac{1}{f_{\text{desired}}}-\frac{1}{n_{\text{strokes}}}\sum_{s=1}^{n_{\text{strokes}}}\left(T_{\text{played},s}\right)\right|/s\right)\text{ and} \end{array}$$

(11) $$\begin{array}{l} y_{\text{ln}(\text{imprecision})}=\ln\left(\underset{s\in \left\{1,\ldots ,n_{\text{strokes}}\right\}}{SD}(T_{\text{played},s})/s\right). \end{array}$$

Hence, these transformed outcome measures were used in the statistical analysis of the experiment. The results of the statistical model were calculated as the numerical values of the parameters of the mixed-effects regression model. The most important effects were plotted as 95% confidence intervals, which allows hypothesis testing at a significance level of α = 0.05.

## 3. Results

The measurement data is summarized in Table 2. The measured inaccuracy ranges from less than 0.1 to 39 ms, the imprecision from 4 to 33 ms, the perceived stress from 1 to 8 (the whole range of the questionnaire) and the normalized muscular activity from 0.36 to 2.76. Comparing the imprecision at the highest desired frequency to the results of Fujii et al. (2009, Table 3), the experts in Fujii et al. (2009) play slightly better, while their novices play slightly worse.

**Table 2:** Summary of the measurement data.

	**Minimum**	**1st quartile**	**Median**	**Mean**	**3rd quartile**	**Maximum**
Inaccuracy (ms)	<0.1	1.58	2.12	4.58	3.36	39.2
Imprecision (ms)	4.26	7.51	9.26	11.2	13.5	33.2
Perceived stress	1	2	4	4.3	6	8
Muscular activity	0.36	0.72	0.87	0.92	1.05	2.76

*Note that for the statistical model, the values inaccuracy and imprecision were transformed by the natural logarithm*.

*Here the values are shown untransformed for easier interpretation*.

**Table 3:** Comparison of imprecision (Equation 2) at maximum drumming frequency between Fujii et al. (2009) and our study (mean±SD).

	**Fujii et al., 2009**	**Our study at a desired frequency of 6.3 Hz**				
	**Own hand**	**Spring 1**	**Spring 2**	**Spring 3**	**Spring 4**	**Stiff conn.**
Novices	27 ± 20 ms at 7.0 ± 0.9 Hz	18 ± 10 ms	14 ± 6 ms	18 ± 7 ms	20 ± 9 ms	12 ± 3 ms
Experts	7 ± 5 ms at 6.8 ± 0.6 Hz	14 ± 8 ms	13 ± 7 ms	13 ± 7 ms	13 ± 4 ms	9 ± 3 ms

Fitting the statistical models to the measurement data yields values for the parameters that tell how much each of the factors influenced the outcome measures. Values for all fitted parameters are found in Table 4.

**Table 4:** Estimates for the parameters of the fitted linear mixed models in Equation (7).

	**Estimated effects on outcome measures**			
**Parameter**	**ln(inaccuracy)**	**ln(imprecision)**	**Perceived stress**	**Muscular activity**
β_0_	−6.094	−4.895	2.845	0.925
β_adapter_type,1_	0.218	0.361	0.594	−0.165
β_adapter_type,2_	0.311	0.296	0.238	−0.231
β_adapter_type,3_	0.020	0.222	0.224	−0.209
β_adapter_type,4_	−0.046	0.147	0.872	−0.153
β_desired_frequency,1_	0.334	0.028	0.694	0.077
β_desired_frequency,2_	0.472	0.126	1.709	0.173
β_desired_frequency,3_	0.678	0.129	2.945	0.559
β_expert_status,1_	0.471	0.339	−1.143	−0.082
β_diagonal,1_	−0.358	0.074	−0.183	0.043
β_trial_number_	−0.030	0.000	−0.022	−0.006
β_diagonal×expert_status,11_	0.693	0.091	0.150	−0.055
β_adapter_type×expert_status,11_	0.169	0.094	0.872	0.194
β_adapter_type×expert_status,21_	0.201	−0.028	0.619	0.113
β_adapter_type×expert_status,31_	0.102	0.036	0.320	0.162
β_adapter_type×expert_status,41_	0.263	−0.024	−0.097	0.116

The inaccuracy is most strongly influenced by the expert status and the desired frequency. The imprecision is most strongly influenced by the adapter type and expert status. The perceived stress is dominated by the desired frequency. The most important influence factors on muscular activity are desired frequency and adapter type.

The effect of the trial number, which represents learning and fatigue, is between one and three orders of magnitude smaller than the other effects.

**Figure 6:**
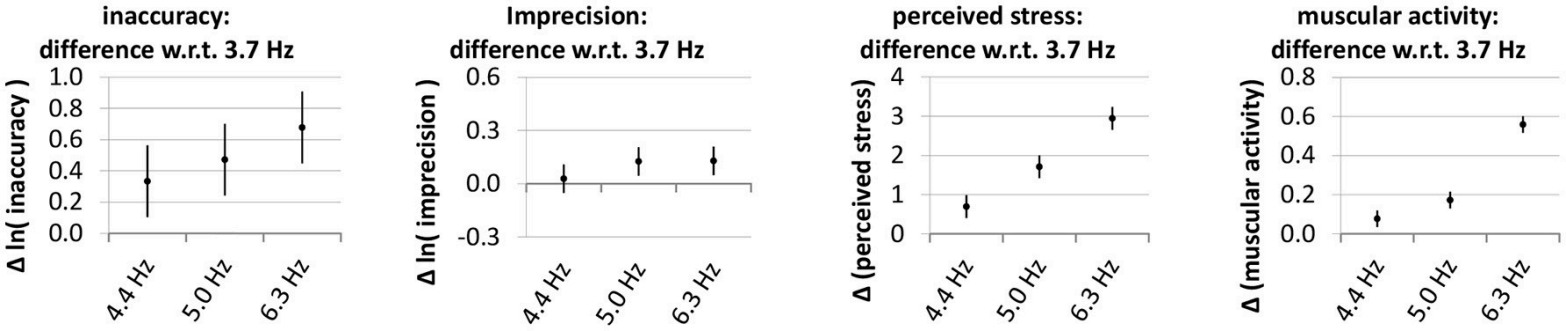
Effect of the desired frequency on the outcome measures. The diagram shows the estimates and 95% confidence intervals of the differences between outcome measures at the higher desired frequencies and the outcome values at a frequency of 3.7 Hz. Since the statistical model (Equation 7) does not contain any interaction terms between frequency and expert status, the values are the same for experts and novices. Lower values are better.

**Figure 7:**
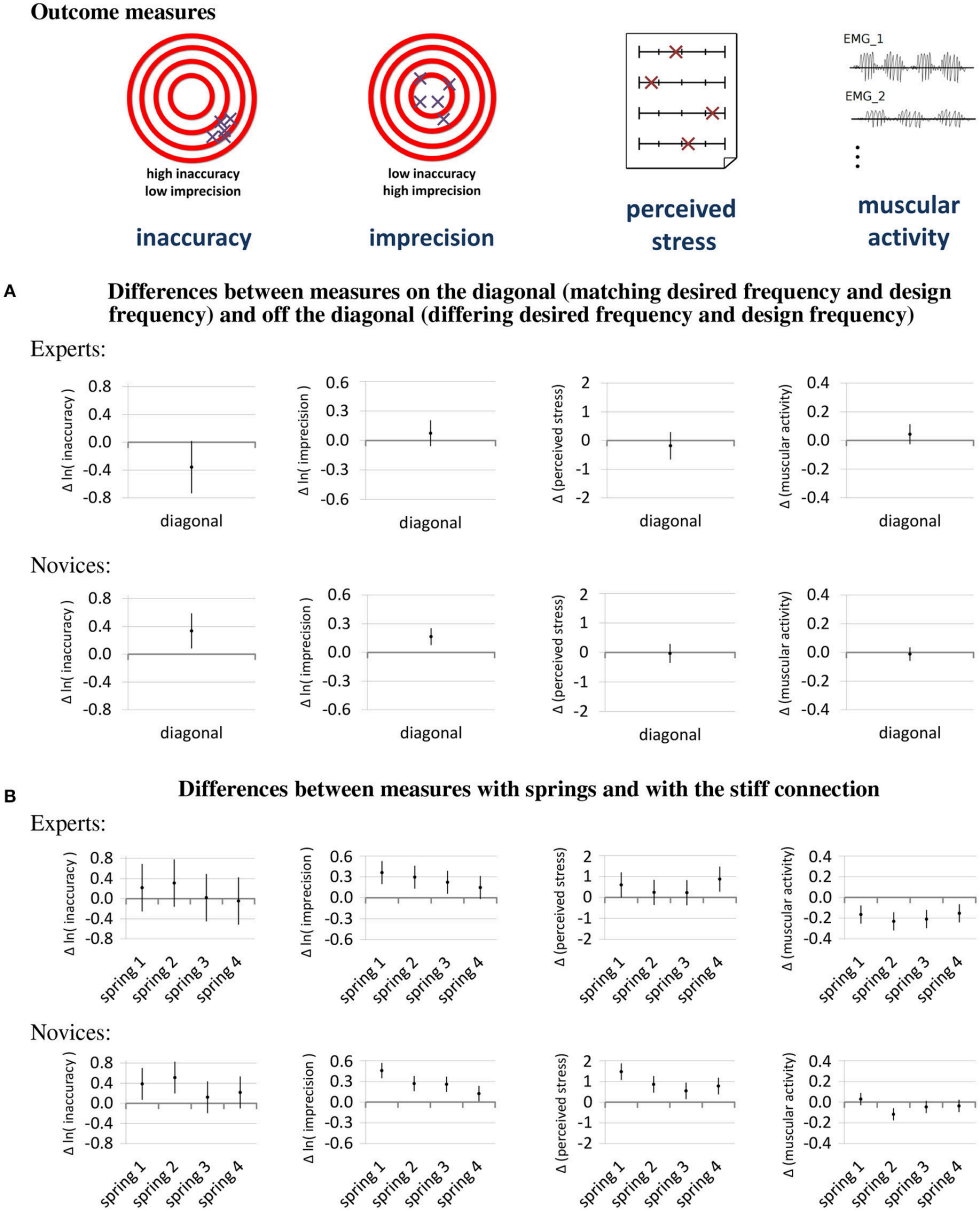
Main results— The diagrams in this figure show how the four outcome measures are affected by the factors diagonal **(A)** and adapter type **(B)**. The dots in the diagram represent the estimates of the effects on the measures and the lines represent their 95% confidence intervals. For all outcome measures, lower values are better.

**Figure 8:**
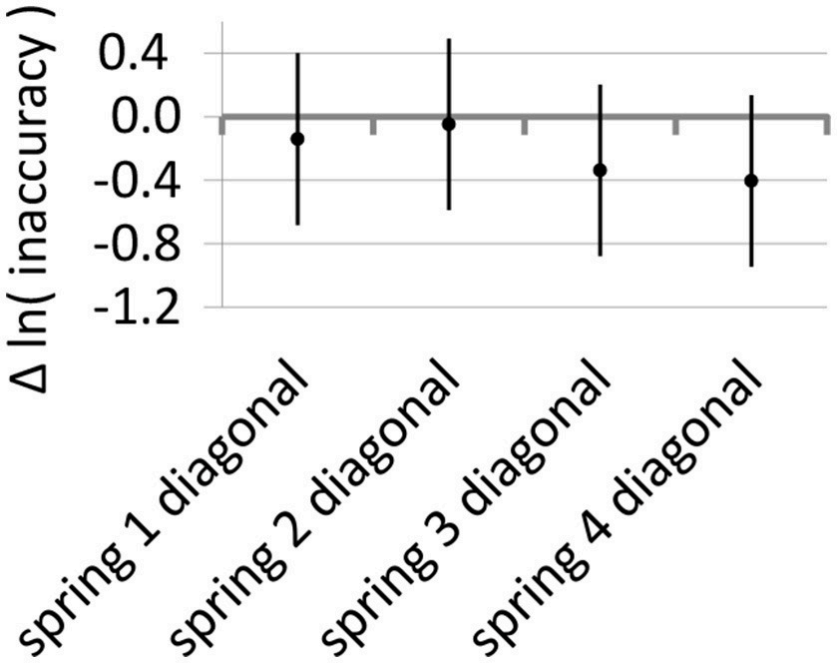
Effect of variable stiffness on the inaccuracy of experts. The diagram shows the estimates and 95% confidence intervals of the differences between inaccuracy with diagonal-type variable-stiffness springs and with the rigid connection. Lower values are better.

The effects of the most important parameters on the outcome measures are shown as 95% confidence intervals in Figures 6–**8**. Whenever the 95% confidence interval does not include zero, the effect is statistically significant at a significance level of α = 0.05. In some cases of a slight overlap between the confidence interval and the zero level we carefully speak of *tendencies*.

Figure 6 depicts the influence of the desired frequency on the outcome measures. On all of them, increasing the desired frequency has a detrimental effect.

The influence of the factors that are related to the question which adapter type is most suitable for a drumming prosthesis, namely the adapter type, the diagonal status (i.e., whether the design frequency matches the desired frequency) and their interactions with the expert status, are shown in Figure 7.

The first hypothesis, that variable stiffness provides the best performance and comfort, and the second hypothesis, that experts are better able to make use of the variable stiffness, can be tested by regarding the differences between playing on or off the diagonal shown in Figure 7A. The effects of the diagonal on the inaccuracy are in opposite directions for experts and novices^2^. For the experts, the effect of the diagonal is a reduction of the inaccuracy, while for novices it is an increase in inaccuracy. The imprecision of novices also increases with playing on the diagonal and the imprecision of experts shows a trend of increase. The comfort measures were less affected by the diagonal.

Figure 7B shows the differences between playing with an elastic spring and playing with the stiff connection and helps to answer the question whether stiff or elastic adapters are more suitable. Regarding performance, the springs show a mostly detrimental effect. The imprecision when playing with the springs is higher than when playing with the stiff connection and increases with increasing softness of the springs. The inaccuracy also shows a tendency to increase when using springs instead of a stiff connection, especially for the springs with lower stiffness. Regarding the influence of using elastic springs on the comfort measures, there is a discrepancy between perceived stress and muscular activity. On the one hand, the perceived stress of novices is higher when using the springs, and also the perceived stress of experts tends to be higher. On the other hand, playing with springs rather than a stiff connection reduces the muscular activity of experts and shows a tendency for reduction of the muscular activity of novices.

Since the use of diagonal-type variable stiffness shows a beneficial effect on the accuracy of experts but the use of some of the springs shows a detrimental effect with a similar magnitude, it is interesting to see the differences in inaccuracy of experts between using the springs in diagonal-type variable stiffness mode and using the stiff connection. These effects of the springs on the diagonal are the sum of the effect of the diagonal (Figure 7A) and the effects of the springs (Figure 7B) and are shown in Figure 8. The diagram shows a tendency for reduced inaccuracy when playing with diagonal-type variable stiffness.

## 4. Discussion and conclusion

In this study, we investigated whether drummers can take advantage of a variable-stiffness joint in a prosthetic wrist. We asked 20 novices and 9 experts to play different frequencies using different elastic elements and one stiff element in a connection between a cuff on the forearm and a drumstick. We hypothesized that subjects will perform best and require the least effort when playing the elastic elements at their resonant frequencies, which is one main argument for variable-stiffness actuation. Moreover, we hypothesized that such an effect will be more obvious for an expert drummer.

*Can drummers take advantage of a variable-stiffness joint in a prosthetic wrist?* Our experimental design was unable to verify that variable stiffness is useful for a prosthetic wrist. Even if experts showed a trend for a reduced inaccuracy when playing the diagonal, they showed the opposite trend for imprecision, and it is difficult to judge which of the two is more relevant. For novices, both inaccuracy and imprecision increased when using the diagonal-type variable stiffness.

The results showed the expected influence of elasticity on the EMG-measured effort, namely that elasticity reduces the effort, and that experts are better than novices in doing so. Despite that, there was a clear discrepancy between measured and perceived effort for both groups. While the muscular activity decreased with decreasing stiffness of the adapter, the perceived stress increased. We find this effect surprising.

A possible interpretation is that subjects are indeed able to save muscular effort by making use of the energy-storing capabilities of the spring. However, the effort by the brain for controlling the more complex dynamics of the system involving the springs is likely higher than for controlling the system involving the stiff connection. In the answers to the questionnaire, the increased cerebral effort might therefore outweigh the reduced muscular effort.

A further explanation for the lower EMG-measured effort when using the springs lies in the fact that the they are more lightweight than the stiff connection and that less muscular effort might have been necessary to accelerate and decelerate it. However, the reduction of inertia (5–6%) is considerably lower than the reduction of EMG-measured effort (16–25%). Assuming that muscular activity is roughly proportional to accelerated inertia, we therefore estimate that the influence of the different weights on the EMG-measured effort is small and that the effect of the adapter types on muscular activity is dominated by their elasticity.

These findings somewhat confirm the study of Fujisawa and Miura (2010), who found that removing the rebound of the drum or adding weight would lead to increased EMG levels. However, this comparison is limited by differing places of energy storage (drum skin vs. elastic wrist) and investigated muscles (wrist muscles vs. elbow and shoulder muscles).

In interpreting the results with respect to variable stiffness, three main limitations of our experimental design have to also be considered. (a) We measured the resonant frequency of the cuff including the elastic element and the drumstick. The influence of the rebound of the drum skin as well as the elasticity of the soft tissue and the (variable) stiffness of the joints due to muscle contraction and cocontraction were not gathered when measuring the resonant frequency of the cuff-spring-drumstick combination. This possibly leads to lower resonant frequencies of the whole system including the human arm, which could explain the better performance of the stiffer springs. (b) There might be variable-stiffness strategies other than matching the resonant frequency of the system to the desired frequency. In order to potentially discover those other variable-stiffness strategies, in future experiments, one could ask subjects to play a more fine grained set of frequencies and try to discover patterns in the outcome measures. (c) Even the expert drummers were naive to the experiment and the time to become acquainted with the system for any combination of adapter type and desired frequency was limited to 10 s. While this was helpful for achieving a reasonable experiment duration, allowing a subject to practice using a variable-stiffness drumming system for weeks or months might show long-term learning effects.

Future experiments into variable-stiffness drumming prostheses might use the following improvements to possibly find a beneficial effect of variable stiffness. A new device with continuously variable stiffness instead of separate adapters with different stiffness levels could be built so that subjects can better match the resonant frequency of the whole system including the rebound of the drum to the desired frequency or employ a different variable-stiffness strategy. This would also remove the problem of different masses of the stiff connection adapter and the springs. Furthermore, subjects could be given more time (hours, days or weeks) to become more acquainted with the system in order to learn the more complex dynamics.

Conclusively, our experimental results argue that series-elastic elements can be used to reduce the muscular activity of drumming, but that their stiffness does not need to be variable. However, the elastic elements appear to initially put an increased control burden on the user. While expert drummers seem to be able to deal with their more complex dynamics, novice drummers seem to reach a better performance with a stiff connection. Prosthesis users may benefit from this study if its results or the results of a future, improved study are incorporated into the design of an actual prosthetic device.

## Author contributions

GS, HH, and JV were involved in planning and design of the experiments. HH and GS were involved in the statistical analysis. JS was involved in setting up and conducting the experiments. HN, HH, and GS were involved in the statistical design. HH and GS wrote the manuscript. JV provided corrections to the manuscript. All authors contributed to manuscript revision, read and approved the submitted version.

### Conflict of interest statement

The authors declare that the research was conducted in the absence of any commercial or financial relationships that could be construed as a potential conflict of interest. The reviewer NC and handling Editor declared their shared affiliation.

## Footnotes

^1^This is translated from the original questionnaire, which was in German: “Wie hoch war die empfundene Belastung beim Spielen der einzelnen Frequenzen, in Bezug auf körperliche und geistige Anstrengung” 1: sehr wenig, 8: sehr viel.

^2^Since the logarithm is a monotonically increasing function, a higher logarithmic outcome measure corresponds to a higher plain outcome measure, so that, for example, a higher logarithmic inaccuracy can be described simply as a higher inaccuracy.

## Abbreviations

DC: Direct current, static part of a signal
DLR: Deutsches Zentrum für Luft- und Raumfahrt, German Aerospace Center
EMG: electromyography
LMU: Ludwig-Maximilians-Universität
RMS: square root of mean squared
SD: standard deviation
SEA: series-elastic actuator
VSA: variable-stiffness actuator.

## References

[B1] Ajoudani A. Tsagarakis N. G. Bicchi A. (2012). Tele-impedance: teleoperation with impedance regulation using a body-machine interface. Int. J. Rob. Res. 31, 1642–1656. 10.1177/0278364912464668

[B2] Albu-Schäffer A. Haddadin S. Ott C. Stemmer A. Wimböck T. Hirzinger G. (2007). The DLR lightweight robot: design and control concepts for robots in human environments. Indt. Rob. Int. J. 34, 376–385. 10.1108/01439910710774386

[B3] Albu-Schäffer A. Hirzinger G. (2002). Cartesian impedance control techniques for torque controlled light-weight robots, in Robotics and Automation, 2002. Proceedings. ICRA '02. IEEE International Conference on, Vol. 1 (Washington, DC), 657–663.

[B4] Bates D. Mächler M. Bolker B. Walker S. (2015). Fitting linear mixed-effects models using lme4. J. Stat. Softw. 67, 1–48. 10.18637/jss.v067.i01

[B5] Bäuml B. Hirzinger G. (2008). When hard realtime matters: Software for complex mechatronic systems. Rob. Auton. Syst. 56, 5–13. 10.1016/j.robot.2007.09.017

[B6] Bretan M. Gopinath D. Mullins P. Weinberg G. (2016). A robotic prosthesis for an amputee drummer. arXiv preprint arXiv:1612.04391.

[B7] De Serres S. J. Milner T. E. (1991). Wrist muscle activation patterns and stiffness associated with stable and unstable mechanical loads. Exp. Brain Res. 86, 451–458. 10.1007/BF00228972 1756819

[B8] Fujii S. Kudo K. Ohtsuki T. Oda S. (2009). Tapping performance and underlying wrist muscle activity of non-drummers, drummers, and the world's fastest drummer. Neurosci. Lett. 459, 69–73. 10.1016/j.neulet.2009.04.055 19409958

[B9] Fujisawa T. Miura M. (2010). Investigating a playing strategy for drumming using surface electromyograms. Acoust. Sci. Technol. 31, 300–303. 10.1250/ast.31.300

[B10] Godfrey S. B. Ajoudani A. Catalano M. Grioli G. Bicchi A. (2013). A synergy-driven approach to a myoelectric hand, in IEEE International Conference on Rehabilitation Robotics (Seattle, WA), 1–6. 10.1109/ICORR.2013.6650377 24187196

[B11] Grebenstein M. Albu-Schäffer A. Bahls T. Chalon M. Eiberger O. Friedl W. (2011). The DLR hand arm system, in IEEE International Conference on Robotics and Automation (ICRA) (Shanghai), 3175–3182.

[B12] Hajian A. Z. (1997). A Characterization of the Mechanical Impedance of Human Hands. Ph.D. thesis, Harvard.

[B13] Hajian A. Z. Sanchez D. S. Howe R. D. (1997). Drum roll: increasing bandwidth through passive impedance modulation, in Proceedings of International Conference on Robotics and Automation, Vol. 3 (Albuquerque, NM), 2294–2299.

[B14] Hocaoglu E. Patoglu V. (2012). Tele-impedance control of a variable stiffness prosthetic hand, in 2012 IEEE International Conference on Robotics and Biomimetics (ROBIO) (Guangzhou), 1576–1582.

[B15] Höppner H. Lakatos D. Urbanek H. Castellini C. van der Smagt P. (2011). The Grasp Perturbator: calibrating human grasp stiffness during a graded force task, in Proceedings of IEEE International Conference on Robotics and Automation (ICRA), 2011 (Shanghai), 3312–3316.

[B16] Höppner H. Stillfried G. Große-Dunker M. Bayer J. van der Smagt P. (2017). Key insights into hand biomechanics: Human Grip Stiffness Can Be Decoupled from Force by Cocontraction and Predicted from Electromyography. Front. Neurorobot. 11:17. 10.3389/fnbot.2017.00017 28588472 PMC5438998

[B17] Kuchenbecker K. J. Park J. G. Niemeyer G. (2003). Characterizing the human wrist for improved haptic interaction, in Proceedings of ASME Proceedings Mechanical Engineering Congress and Exposition, Vol. 2 (Washington, DC), 42017.

[B18] Lakatos D. Petit F. Albu-Schäffer A. (2014). Nonlinear oscillations for cyclic movements in human and robotic arms. IEEE Trans. Robot. 30, 865–879. 10.1109/TRO.2014.2308371

[B19] Latash M. L. Zatsiorsky V. M. (1993). Joint stiffness: myth or reality? Hum. Mov. Sci. 12, 653–692. 10.1016/0167-9457(93)90010-M

[B20] Lwp Kommunikáció (2014). Photo Jason Barnes. Available online at: https://www.flickr.com/photos/lwpkommunikacio/15579354269 (Accessed August 3, 2017).

[B21] R Core Team (2015). R: A Language and Environment for Statistical Computing. Available online at: https://www.R-project.org/ (Accessed January 5, 2018).

[B22] Sinkjær T. Hayashi R. (1989). Regulation of wrist stiffness by the stretch reflex. J. Biomech. 22, 1133–1140. 10.1016/0021-9290(89)90215-7 2625413

[B23] TRS Inc (2017). Music – Drum Stick. Available online at: http://www.trsprosthetics.com/product/music-drum-stick/ (Accessed December 11, 2017).

[B24] Vanderborght B. Albu-Schäffer A. Bicchi A. Burdet E. Caldwell D. G. Carloni R. (2013). Variable impedance actuators: a review. Robot. Auton. Syst. 61, 1601–1614. 10.1016/j.robot.2013.06.009

[B25] Visser L. Stramigioli S. Bicchi A. (2011). Embodying desired behavior in variable stiffness actuators, in Proceedings of the 18th IFAC World Congress, 2011 (Milan: IFAC), 9733–9738.

